# Impact of the introduction of organised screening for cervical cancer in Turin, Italy: cancer incidence by screening history 1992–98

**DOI:** 10.1038/sj.bjc.6602705

**Published:** 2005-07-12

**Authors:** G Ronco, S Pilutti, S Patriarca, G Montanari, B Ghiringhello, R Volante, L Giordano, R Zanetti, E Mancini, N Segnan, and the Turin Cervical Screening Working Group

**Affiliations:** 1 Unit of Cancer Epidemiology, Centre for cancer Epidemiology and Prevention (CPO Piemonte), via San Francesco da Paola 31, 10123 Torino, Italy; 2 Unit of Pathology, OIRM S Anna, Cso Spezia 60, Torino, Italy; 3 Centre for early cancer diagnosis and treatment, OIRM S Anna, Cso Spezia 60, Torino, Italy

**Keywords:** cervical cancer, screening, organised programme, effectiveness

## Abstract

After an organised cervical screening programme was introduced in Turin in 1992, the age-adjusted cervical cancer incidence ratio in 1992–98 was 0.81 (95% confidence interval (CI) 0.59–1.09) for invited *vs* not invited women and 0.25 (95% CI 0.13–0.50) for attenders *vs* non attenders. An organised screening programme can further reduce cervical cancer incidence in an area where substantial spontaneous activity was previously present.

There is clear evidence of efficacy for cervical cancer screening (IARC, 2005). High effectiveness and cost-effectiveness were obtained in Nordic countries, where cervical screening was organised from the outset (Laara *et al*, 1987). In the United Kingdom, a relevant reduction in cervical cancer mortality followed reorganisation (Sasieni and Adams, 1999; Peto *et al*, 2004), There is however little experience of the impact of moving from opportunistic activity to an organised programme.

For many years, cervical cancer screening in Italy was almost only opportunistic (Segnan *et al*, 2000). An organised programme started in Turin in 1992, the main changes being (a) an active call–recall system, (b) protocols for diagnosis and treatment, (c) a fail-safe system for both these phases and (d) monitoring and intensive quality assurance for every step of the screening process.

In order to evaluate the impact of these changes on invasive cervical cancer incidence, we linked the screening registry to the local population cancer registry.

## MATERIALS AND METHODS

From June 1992, female Turin inhabitants aged 25–64 years were invited, irrespective of their previous spontaneous screening history, for a Pap-test, with 3-year intervals for screen-negatives. Activation was progressive: in 1996, about 78% of the target population had been invited and in 1998, about 95%. The order of invitation was substantially random, with some stratification by area (women were enrolled by general practitioners, who were selected with balance by location of practice).

About one-third of invited women attended the organised programme. Attenders mostly had not had spontaneous recent cytology. As a combined result of organised and opportunistic screening, we estimated a 74% 3-year overall coverage (Ronco *et al*, 1997) *vs* 40% before the start of the programme (Segnan *et al*, 1990).

We computed cervical cancer incidence for women aged 24–69 years in the following groups:

- not invited (not yet having received the first invitation in the organised programme),
- invited (having already received the first invitation),
- attenders (invited women with at least one cytology in the organised programme),
- nonattenders (invited women with no cytology in the organised programme).

Person-years (py) at risk of cervical cancer for the last three categories were calculated using the computerised screening registry. It was not possible to consider the screening history outside the organised programme. Each woman contributed to the ‘invited’ cohort from first invitation to the end of follow-up, that is, the earliest among (a) diagnosis of cervical cancer, (b) death or emigration, (c) 70th birthday or (d) 31 December 1998 (end of study). Person-years for attenders were computed from first cytology within the organised programme to the end of follow-up. Person-years for uninvited women were computed as difference between the Turin female population in each calendar year (estimated on 30 June) and those contributing to the ‘invited’ in the same year.

Invasive cancers of the uterine cervix diagnosed in Turin inhabitants from 1992 to the end of 1998 were obtained from the Piedmont Cancer Registry. Cancers arising in attenders were further classified as diagnosed after a normal cytology (repeat recommended after 3 years) or after a non-normal cytology (any other recommendation, therefore including repeats for unsatisfactory smears).

Cancers reported as microinvasive (FIGO stage 1A1) and those not classified as squamous cell carcinoma (‘adenocarcinoma’ or ‘other specified morphology’ according to Parkin *et al* (1998) (56 cases) were excluded. We included 14 cases with unspecified morphology to maximise power and reduce possible bias due to lack of a histological examination or to insufficient reporting, as both of these could be associated with screening history and their inclusion prevents bias.

Incidence rates, standardised in 5-year age groups on the world population (truncated to the 24–69 age), were calculated. Age-adjusted incidence density ratios (IDR) were computed by Poisson regression. The proportion of cancers attributable to noninvitation in the general population, to noncompliance among invited women and to a screening interval longer than 3.5 years among women with previous normal cytology was computed from the above-mentioned age-standardised rates as (*I*_p_–*I*_0_)/*I*_p_, where *I*_p_ is the incidence in the entire relevant population (Turin inhabitants, invited and attenders with ‘negative’ cytology, respectively) and *I*_0_ is the incidence in ‘nonexposed’ women (invited, attenders and attenders within 3.5 years from a negative cytology, respectively).

## RESULTS

Overall, 190 cancers (176 squamous and 14 unspecified) were included in the analysis. During the study period, 254 132 women received at least one invitation in the organised screening programme (median follow-up after first invitation 3.9 years). Among invited women, 762 223 py (83%) were in the first 3 years after invitation and only 156 639 py after the third year.

Incidence among invited and not invited women and, among the invited, attenders and nonattenders is reported in Table 1. The IDR was 0.81 (95% CI 0.59–1.09) between invited and uninvited women and, among the invited, 0.25 (95% CI 0.13–0.50) between attenders and nonattenders in the organised programme. Some 20% of cancers observed in the study population would have been avoided if all women were invited and 56% of cancers arising among invited women would have been avoided if they had all attended the organised programme.

Among attenders, four cases were diagnosed after a normal cytology (Table 2): two within 3.5 years (raw incidence rate 0.7 per 10^5^) and two after more than 3.5 years (raw incidence 9.0 per 10^5^). Some 65% of cancers observed after negative cytology were attributable to a screening interval longer than 3.5 years.

Among the seven cancers diagnosed after abnormal cytology (Table 2), two were identified as a result of screening, all at the first screening episode. However, five cases arose among women who did not follow some recommend action (three did not repeat cytology, one did not have colposcopy and one missed post-treatment clinical follow-up).

## DISCUSSION

In Turin, an organised cervical screening programme was introduced in a population where some spontaneous screening was already present. Comparing cervical cancer incidence between invited and not invited women provides an estimate of its impact, although underestimated as a result of screening-induced diagnostic lead time. This underestimate was partly but not completely corrected by excluding microinvasive cancers, mainly screen-detected; therefore, long-term impact, however, would be expected to be larger. We observed a 20% incidence reduction among invited *vs* uninvited women, consistent with our previous estimate that 17% of coverage among invited women was due to invitation (Ronco *et al*, 1997). Available data also suggest that, as an effect of the organised programme, some of the women who previously had more frequent spontaneous tests switched to the recommended 3-year interval (Ronco *et al*, 1997), resulting in test savings, therefore plausibly in increased cost-effectiveness.

Cervical screening is still opportunistic in many European countries (IARC, 2005; Antilla *et al*, 2005). A similar impact can reasonably be expected in those that would decide to introduce an organised system.

As the order of invitations was substantially random, risks of cervical cancer among invited and uninvited women are expected to be comparable in the absence of screening.

In Denmark, a RR of 0.67 (95% CI, 0.61–0.73) was observed for women aged 30–59 years in 1963–1982 when comparing counties with and without organised screening (Lynge *et al*, 1989).

Attendance in the organised programme was associated with a more than 70% reduction of cervical cancer incidence. This is not an estimate of the protection provided by screening in the organised programme *vs* no screening at all, as many nonattenders were screened spontaneously. We had estimated that about 60% of noncompliers to invitation had had opportunistic cytology within 3 years (Ronco *et al*, 1997). The observed reduction is therefore larger than expected, assuming similar baseline risk among women attending opportunistic and organised screening and similar protection provided by screening in both settings. We cannot exclude different baseline risks (higher detection rate of *in situ* cervical cancer was found among smears taken in opportunistic screening in Sweden; Gustafsson *et al*, 1995) but our previous data showed that invitation increased coverage, especially among women with middle–low education (Ronco *et al*, 1997), who are usually at higher risk. The alternative hypothesis is that screening in the organised programme was more protective than opportunistic activity. A greater effectiveness of organised than spontaneous activity was observed in Finland (Nieminen *et al*, 1999).

Excluding nonsquamous cancers, the crude incidence of invasive cancers within 3.5 years of a normal cytology in the organised programme (0.7 per 10^5^ py) was very low. Cytology in our situation, therefore, showed good sensitivity, perhaps as a result of the intensive quality assurance programme adopted. However, it is remarkable that eight cervical adenocarcinomas arose within 3.5 years from normal cytology, so that with the low efficacy of cervical screening by cytology in preventing adenocarcinoma (IARC, 2005) these were the most common ‘interval’ cancers.

Remarkably, among attenders, five of the 11 cancers occurred in women who did not follow recommendations. In our population, the largest overall reduction in cervical cancer incidence would be obtained by further increasing attendance in the organised programme and by improving compliance to follow-up. In the UK, with recommended intervals of 3–5 years and coverage within 5 years exceeding 80%, it was estimated that 23.5% of fully invasive cancers under the age of 70 years may be attributed to no screening, 2% to a screening interval over 5 years and 10.5% to inadequate follow-up (Sasieni *et al*, 1996).

## Figures and Tables

**Table 1:** Person-years, number of cervical cancers, incidence density and incidence density ratio (IDR) among not invited and invited women and, within invited, among attenders and nonattenders

	**Person-years**	**Cancer cases^a^**	**Crude incidence (per 10^5^ py)**	**Age-standardised incidence^b^**	**IDR^c^**	**95% CI**
Not invited	1 265 075	118	9.3	8.6	1.0	
Invited	918 862	72	7.8	6.9	0.81	0.59–1.09
Invited nonattenders	570 186	61	10.7	9.5	1.0	
Invited attenders	348 676	11	3.2	3.0	0.25	0.13–0.50

a Cases with morphology specified as nonsquamous or staged as microinvasive excluded.

b Standardised on the world population truncated 24–69 years, per 100 000 py.

c Adjusted for age in 5-year groups by Poisson regression.

**Table 2:** Cervical cancers diagnosed among attenders

	**Person-years**	**Cancer cases^a^**	**Crude incidence (per 10^5^ py)**	**Age-standardised incidence^b^**
After non-normal cytology	30 973	7	22.6	23.0
After normal cytology				
Independently of time	317 702	4	1.3	1.2
Within 3.5 years after last normal cytology	295 414	2	0.7	0.4
Over 3.5 years after last normal cytology	22 288	2	9.0	11.1

a Cases with morphology specified as nonsquamous or staged as microinvasive excluded.

b Standardised on the world population truncated 24–69 years, per 100 000 py.
